# Supplementary material for: Identifying hotspots of greenhouse gas emissions from drained peatlands in the European Union
Source: Nat Commun. 2025 Dec 2;16:10825. doi: 10.1038/s41467-025-65841-6 (PMC12673095; doi:10.1038/s41467-025-65841-6)
Supplement: Supplementary file 1 — Supplementary Information [file 41467_2025_65841_MOESM1_ESM.pdf]

Table S.1 | National peatland land use areas. *An overview of the different land uses (kha) per country. The first 40 columns show peatland land cover based on the land uses on peat soils that were not covered by Witjes et al., 2022 land use map and the other columns are based on the detailed (agricultural) land use map of d'Andrimont et al., 2021, which was the first choice map.*

Detailed Land use	Land use Classes	Albania	Andorra	Austria	Belgium	Bulgaria	Bosnia and Herzegovina	Croatia	Czech republic	Denmark	Estonia	Finland	France	Germany	Greece	Hungary	Iceland	Ireland	Italy	Latvia	Liechtenstein	Lithuania	Luxembourg	Montenegro	Netherlands	North Macedonia	Norway	Poland	Portugal	Romania	Serbia	Slovakia	Slovenia	Spain	Sweden	Switzerland	United Kingdom	Total
		Area [kha]																																				
Barley				0,4	0,1			0,0	0,0	4,5	2,0	34,3	0,2	15,9	0,0	0,8		2,1	0,0	1,5		4,8	0,0		8,3			2,9	0,0	0,2		0,0		0,0	0,7	0,0	0,0	**79,0**
Common wheat				9,2	1,8	1,7		0,1	0,2	4,4	11,4	45,3	4,0	79,6	1,1	45,0		2,3	0,1	24,8		63,3	0,1		7,6			33,3	0,0	11,0		0,0	0,0	0,2	2,2	0,0	0,0	**348,7**
Cropland		4,2	0,0	0,0	0,1	0,0	3,0	0,1	0,0	0,7	0,2	0,0	0,7	0,1	0,1	0,1	17,5	0,1	0,0	0,1	0,1	0,4		0,8	0,0	0,2	79,4	0,1	0,1	0,3	3,1		0,0	0,3	0,7	5,6	18,9	**137,0**
Cropland (grassland)			0,0	3,7	1,7	0,1		0,0	0,2	3,5	6,8	46,1	3,7	60,1	0,3	12,0		7,9	0,1	8,2	0,0	25,3	0,1		25,0		0,0	29,1	0,1	3,2		0,0	0,3	0,1	1,5	0,0	0,0	**239,1**
Dry pulses, vegetables, and flowers				0,2	0,1	0,0		0,0	0,0	0,2	0,8	1,0	0,1	2,1	0,0	0,3		0,0	0,0	2,7		8,4	0,0		0,8			2,5	0,0	0,1		0,0		0,0	0,2			**19,5**
Durum wheat												0,0	0,0		0,1	0,0			0,1	0,0		0,0							0,1	0,0				1,4	0,0			**1,8**
Fodder crops				0,5	0,1	0,1		0,0	0,0	0,3	1,1	1,4	0,3	3,1	0,8	5,4		0,0	0,1	0,7	0,0	1,6	0,0		1,6			1,4	0,2	1,8		0,0	0,0	0,2	0,0		0,0	**20,8**
Fruit trees and berry plantations		0,2					0,0						0,0						0,0					0,1						0,0				0,0		0,0		**0,4**
Maize				9,8	5,5	0,3		0,1	0,1	1,4	1,6	3,7	6,0	171,3	6,8	49,5		0,3	0,1	2,4	0,0	8,2	0,0		35,0			38,3	2,5	13,0		0,0	0,6	13,1	0,6	0,0	0,0	**370,2**
Non-irrigated arable land		10,9	0,0	0,0	0,1		3,5	0,0		0,1	0,0		0,2	0,1	0,0	0,0	3,3	0,1	0,0	0,0	0,1	0,2		0,3	0,0	0,3	1,2	0,0	0,0	0,0	0,7			0,0	0,1	14,8	178,7	**214,8**
Oats				0,1	0,0			0,0	0,0	0,7	1,3	3,2	0,0	2,6	0,0	0,1		0,0	0,0	5,2		10,9	0,0		0,2			9,7	0,3	0,0		0,0		0,1	0,9			**35,3**
Olive groves	Cropland	0,0																	0,0					0,0														**0,0**
Other cereals				0,0	0,0			0,0	0,0	0,0	0,9	0,3	0,0	0,2		0,0		0,0		2,6		7,8			0,0			1,2		0,0		0,0		0,0	0,0			**13,2**
Other non-permanent industrial crops				0,0	0,0								0,1	0,0	1,3	0,0			0,0	0,0		0,0			0,0			0,0	0,0	0,0				0,7				**2,2**
Other root crops				0,0	0,0			0,0		0,0	0,0	0,0	0,0	0,0		0,0				0,0		0,0			0,3			0,0		0,0				0,0	0,0			**0,4**
Potatoes				0,4	0,9			0,0	0,0	1,1	1,2	1,6	0,2	20,0	0,0	0,2		0,1	0,0	1,3		3,9	0,0		33,1			3,4		0,0				0,0	0,2	0,0		**67,4**
Rape and turnip rape				0,2	0,0	0,3		0,0	0,0	1,1	1,4	0,1	0,5	13,5	0,0	3,6		0,1		2,7		14,8	0,0		0,2			10,2	0,0	0,8		0,0		0,0	0,3			**49,8**
Rye				0,2	0,0				0,0	0,8	0,3	0,4	0,0	15,0		0,0		0,1		1,0		6,9	0,0		0,3			15,7	0,0	0,0		0,0		0,0	0,2			**41,0**
Soya				1,4	0,0	0,0		0,0	0,0			0,0	0,3	0,9	0,6	1,5			0,0			0,0			0,0			0,1	0,0	0,4		0,0	0,0	0,2	0,0			**5,4**
Sugar beet				0,5	0,5			0,0	0,0	0,1	0,3	1,0	0,3	4,7	0,0	0,2		0,1	0,0	0,7		1,0			14,6			2,4	0,0	0,0		0,0		0,0	0,1	0,0		**26,5**
Sunflower				1,2	0,0	0,7		0,0	0,0			0,0	0,2	0,4	3,0	16,5			0,0	0,0		0,2	0,0		0,0			0,0	0,0	3,8		0,0		0,0				**26,2**
Triticale				0,0	0,0				0,0	0,0	0,0	0,0	0,0	1,8		0,0		0,0		0,1		0,2			0,0			3,1							0,0			**5,2**
Sub_total		15,3	0,0	27,8	11,0	3,2	6,4	0,4	0,7	18,8	29,3	138,4	16,9	391,4	14,2	135,1	20,7	13,2	0,5	54,1	0,2	158,2	0,2	1,2	127,3	0,4	80,6	153,5	3,5	34,8	3,9	0,1	1,0	16,4	7,5	20,4	197,7	1704,4
Broad-leaved forest		0,6		0,3	0,2	0,0	0,3	0,3		0,1	0,0	0,0	1,6	0,1	0,0	0,0	5,3	1,8	0,0	0,1	0,0	0,5		0,5	0,2	0,0	79,9	0,1	0,0	0,0	6,2			0,0	1,1	1,1	60,3	**213,7**
Coniferous forest		0,0		0,0	0,0	0,0	0,0	0,0	0,0	0,0	0,1		0,2	0,0	0,0	0,0	1,2	0,9	0,0	0,0	0,0	0,1		0,0	0,0	0,0	130,5	0,0	0,0	0,0	0,1	0,0	0,0	0,0	0,6	7,6	21,1	**186,3**
Forestland	Forestland			18,6	10,6	0,4		9,1	6,0	18,0	203,6	1178,7	19,4	123,2	1,0	29,6	0,0	380,2	0,4	75,3	0,0	131,6	0,0		16,2		0,1	287,6	0,6	10,0	0,0	0,2	0,7	1,2	622,3	0,0	0,1	**4002,4**
Mixed forest		0,0		0,0	0,0		0,0	0,0		0,0	0,0		0,0	0,0			0,4	0,0		0,0	0,0	0,1		0,0	0,0	0,0	52,5	0,0		0,0	0,0			0,0	0,2	1,8	0,2	**57,3**
Forestland (low productive)				10,6	6,0	0,1		0,2	9,2	1,9	343,1	4648,0	11,6	136,9	0,0	10,3		39,3	0,1	188,0		147,5	0,0		1,9	0,0	0,2	575,9	0,2	1,5	0,0	0,0	1,4	0,1	2404,6	0,2	92,2	**9885,5**
Wet forestland												3,4																							867,6			**871,0**
Sub_total		0,7	0,0	29,6	16,8	0,5	0,3	9,6	15,2	20,1	546,8	5830,1	32,8	260,2	1,1	39,8	6,9	422,3	0,4	263,4	0,0	279,9	0,0	0,5	18,3	0,0	263,2	863,6	0,8	11,5	6,3	0,2	2,1	1,3	3896,5	10,6	173,9	14345,2
Grassland		1,5	0,0	35,3	24,8	1,9	5,2	2,8	3,8	79,7	66,5	176,8	57,7	650,5	0,8	117,4	282,8	344,1	0,2	41,9	0,4	118,8	0,5	0,3	203,8	0,2	0,5	285,5	0,4	36,4	2,4	0,2	2,6	3,5	42,0	29,8	270,7	**2891,6**
Grassland (low productive)	Grassland	0,0	0,0	9,0	2,9	0,2		0,0	0,8	2,1	13,9	6,8	9,2	303,6	0,1	54,0		87,5	0,2	27,0		41,9	0,1		50,1		0,0	272,4	0,0	3,9	0,0	0,2	0,2	0,4	3,1	0,3	449,0	**1339,0**
Sub_total		1,5	0,0	44,3	27,8	2,1	5,2	2,8	4,6	81,8	80,4	183,6	66,9	954,1	0,8	171,4	282,8	431,6	0,5	68,9	0,4	160,7	0,6	0,3	253,9	0,2	0,5	557,9	0,5	40,3	2,4	0,4	2,8	3,9	45,0	30,1	719,6	4230,6
Mineral extraction sites	Mineral extraction sites	0,1		0,0	0,0	0,0	0,0			0,0	0,0		0,0	0,0		0,0	0,0	0,1	0,0	0,0	0,0	0,0		0,0	0,0	0,0	0,2	0,0		0,0	0,0			0,0	0,0	0,1	5,1	**5,6**
Urban	Urban	0,3		11,4	9,5	0,6	0,1	0,1	0,3	1,4	3,8	9,6	3,8	55,2	0,6	18,2	0,5	19,0	0,3	3,7	0,0	4,5	0,1	0,0	57,7	0,0	1,8	16,5	0,4	4,1	0,0	0,0	0,4	8,0	13,3	4,3	16,9	**266,6**
Water			0,0	0,5	0,8	0,1		0,3	0,2	0,6	1,6	2,6	1,6	6,1	0,2	5,2		1,4	0,3	2,5	0,0	5,1	0,0		2,9		0,0	7,9	0,0	4,2		0,0	0,0	0,7	2,7	0,0	0,0	**47,7**
Water		0,4	0,0	2,6	1,4	1,0	1,1	0,7	0,4	1,3	1,8	14,2	5,0	10,0	1,0	17,6	0,5	8,0	3,0	1,3	0,0	4,1	0,0	0,9	10,6	0,0	7,3	7,6	0,3	32,2	0,2	0,0	0,0	2,5	2,3	1,2	26,9	**167,3**
Wet grassland	Wetland											2,9																							181,8			**184,7**
Wetland		3,2	0,4	12,6	7,1	2,9	2,6	4,8	1,1	34,1	179,0	2715,4	50,8	92,1	4,5	81,2	743,0	763,7	5,3	117,8	0,0	56,8	0,0	3,1	21,4	2,1	2168,9	117,8	1,9	206,3	2,1	0,6	0,1	26,1	3007,2	12,8	3723,3	**14172,3**
Sub_total		3,6	0,5	15,8	9,3	4,0	3,7	5,8	1,6	36,0	182,4	2735,1	57,3	108,2	5,8	104,0	743,5	773,1	8,6	121,6	0,0	66,0	0,0	4,0	34,9	2,2	2176,3	133,3	2,2	242,7	2,3	0,6	0,1	29,3	3193,9	14,0	3750,1	14572,1
Total		**22,1**	**0,5**	**166,3**	**106,8**	**11,4**	**16,3**	**22,4**	**29,6**	**218,3**	**1076,1**	**8896,8**	**219,5**	**2066,8**	**23,5**	**543,7**	**1054,5**	**1659,2**	**11,5**	**954,4**	**0,7**	**873,9**	**1,5**	**6,7**	**525,7**	**2,9**	**2522,7**	**2269,5**	**8,6**	**354,6**	**16,1**	**2,3**	**7,6**	**62,9**	**7156,1**	**89,5**	**4994,6**	**35995,6**

Table S.2 | Agricultural peatland area and emissions. The absolute and cumulative fraction of total EU+ area and emissions of agricultural land (Grassland and Cropland) on peat soils. The countries above the green line area have similar (±20%) area and emissions in this study and in the NIS 2023.

	Agricultural crops (Grassland & Cropland)																	
	NIS 2023			Martin and Couwenberg			This Study			NIS 2023			Martin & Couwenberg			This study		
Country	Area [kha]	%	%cum	Area [kha]	%	%cum	Area [kha]	%	%cum	Emissions [kt]	%	%cum	Emissions [kt]	%	%cum	Emissions [kt]	%	%cum
Germany	1296	25,8	25,78	1313	21,5	21,5	1346	22,6	22,61	44165	37,8	37,81	38419	23,2	23,23	37609	22,7	22,69
Netherlands	341	6,8	32,56	338	5,5	27,0	381	6,4	29,0	6220	5,3	43,1	9685	5,9	29,1	11213	6,8	29,5
Finland	342	6,8	39,37	329	5,4	32,4	322	5,4	34,4	9839	8,4	51,6	9204	5,6	34,7	9790	5,9	35,4
Iceland	347	6,9	46,27	-	0,0	32,4	304	5,1	39,5	7898	6,8	58,3	-	0,0	34,7	8736	5,3	40,6
Norway	70	1,4	47,67	-	0,0	32,4	81	1,4	40,9	2463	2,1	60,4	-	0,0	34,7	2954	1,8	42,4
Serbia	-	0,0	47,67	-	0,0	32,4	6	0,1	41,0	-	0,0	60,4	-	0,0	34,7	209	0,1	42,5
Czech republic	0	0,0	47,67	4	0,1	32,5	5	0,1	41,1	0	0,0	60,4	114	0,1	34,7	148	0,1	42,6
Bulgaria	3	0,1	47,73	41	0,7	33,2	5	0,1	41,2	98	0,1	60,5	1364	0,8	35,5	174	0,1	42,7
Slovenia	4	0,1	47,80	9	0,1	33,3	4	0,1	41,2	97	0,1	60,6	292	0,2	35,7	111	0,1	42,8
Portugal	0	0,0	47,80	26	0,4	33,8	4	0,1	41,3	0	0,0	60,6	834	0,5	36,2	139	0,1	42,9
Croatia	3	0,1	47,86	5	0,1	33,8	3	0,1	41,4	107	0,1	60,7	161	0,1	36,3	94	0,1	42,9
North Macedonia	-	0,0	47,86	-	0,0	33,8	2	0,0	41,4	-	0,0	60,7	-	0,0	36,3	22	0,0	43,0
Luxembourg	0	0,0	47,86	0	0,0	33,8	1	0,0	41,4	0	0,0	60,7	6	0,0	36,3	23	0,0	43,0
Slovakia	0	0,0	47,86	12	0,2	34,0	1	0,0	41,4	0	0,0	60,7	368	0,2	36,6	13	0,0	43,0
Montenegro	-	0,0	47,86	-	0,0	34,0	1	0,0	41,4	-	0,0	60,7	-	0,0	36,6	52	0,0	43,0
Liechtenstein	0	0,0	47,86	-	0,0	34,0	1	0,0	41,4	0	0,0	60,7	-	0,0	36,6	0	0,0	43,0
Andorra	-	0,0	47,86	-	0,0	34,0	0	0,0	41,4	-	0,0	60,7	-	0,0	36,6	0	0,0	43,0
Cyprus	0	0,0	47,86	0	0,0	34,0	0	0,0	41,4	0	0,0	60,7	0	0,0	36,6	0	0,0	43,0
United Kingdom	676	13,4	61,29	761	12,5	46,5	917	15,4	56,8	11981	10,3	70,9	14756	8,9	45,5	22216	13,4	56,4
Poland	957	19,0	80,33	922	15,1	61,6	711	12,0	68,8	8633	7,4	78,3	26339	15,9	61,4	18123	10,9	67,4
Ireland	339	6,7	87,07	333	5,5	67,1	445	7,5	76,3	8954	7,7	86,0	5892	3,6	65,0	11630	7,0	74,4
Lithuania	127	2,5	89,60	283	4,6	71,7	319	5,4	81,6	724	0,6	86,6	6091	3,7	68,7	9806	5,9	80,3
Hungary	0	0,0	89,60	56	0,9	72,6	307	5,1	86,8	0	0,0	86,6	2018	1,2	69,9	9123	5,5	85,8
Latvia	81	1,6	91,22	158	2,6	75,2	123	2,1	88,8	2867	2,5	89,1	5054	3,1	72,9	3597	2,2	88,0
Denmark	169	3,4	94,58	179	2,9	78,1	101	1,7	90,5	5282	4,5	93,6	6115	3,7	76,6	2971	1,8	89,8
Estonia	41	0,8	95,39	246	4,0	82,2	110	1,8	92,4	796	0,7	94,3	4450	2,7	79,3	3174	1,9	91,7
France	13	0,3	95,65	139	2,3	84,4	84	1,4	93,8	21	0,0	94,3	4557	2,8	82,1	2395	1,4	93,1
Romania	8	0,2	95,81	626	10,3	94,7	75	1,3	95,0	229	0,2	94,5	20019	12,1	94,2	2359	1,4	94,5
Austria	13	0,3	96,07	100	1,6	96,3	72	1,2	96,2	376	0,3	94,8	3388	2,0	96,2	2156	1,3	95,8
Sweden	147	2,9	98,99	165	2,7	99,0	53	0,9	97,1	4039	3,5	98,3	4384	2,7	98,9	1281	0,8	96,6
Switzerland	17	0,3	99,33	-	0,0	99,0	51	0,8	98,0	682	0,6	98,9	-	0,0	98,9	1591	1,0	97,6
Belgium	3	0,1	99,38	15	0,3	99,3	39	0,7	98,6	89	0,1	98,9	434	0,3	99,1	1149	0,7	98,3
Spain	0	0,0	99,38	13	0,2	99,5	20	0,3	99,0	0	0,0	98,9	330	0,2	99,3	705	0,4	98,7
Albania	-	0,0	99,38	-	0,0	99,5	17	0,3	99,3	-	0,0	98,9	-	0,0	99,3	605	0,4	99,1
Greece	7	0,1	99,52	7	0,1	99,6	15	0,3	99,5	280	0,2	99,2	248	0,1	99,5	540	0,3	99,4
Albania	-	0,0	99,52	-	0,0	99,6	17	0,3	99,8	-	0,0	99,2	-	0,0	99,5	605	0,4	99,8
Bosnia and Herzegovina	-	0,0	99,52	-	0,0	99,6	12	0,2	100,0	-	0,0	99,2	-	0,0	99,5	381	0,2	100,0
Italy	24	0,5	100,00	23	0,4	100,0	1	0,0	100,0	972	0,8	100,0	840	0,5	100,0	29	0,0	100,0
**Total**	**5028**	**100,0**	**100,00**	**6103**	**100,0**	**100,0**	**5952**	**100,0**	**100,00**	**116811**	**100,0**	**100,00**	**165364**	**100,0**	**100,00**	**165722**	**100,0**	**100,00**

Table S.3 | Forest peatland area and emissions. *The absolute and cumulative fraction of total EU+ area and emissions of Forest Land on peat soils. The NIS area data from the countries above the green line roughly agree (±20%) with this research.*

	Forestland											
	NIS 2023			This Study			NIS 2023			This study		
Country	Area [kha]	%	%cum	Area [kha]	%	%cum	Emissions [kt]	%	%cum	Emissions [kt]	%	%cum
**Finland**	5963	45.5	45.48	5827	45.1	45.1	10653	39.2	39.2	18214	16.4	16.4
**Sweden**	4433	33.8	79.29	3897	30.2	75.3	6915	25.5	64.7	21997	19.8	36.2
**Estonia**	288	2.2	81.49	547	4.2	79.5	1052	3.9	68.5	10088	9.1	45.2
**Ireland**	414	3.2	84.65	422	3.3	82.8	2998	11.0	79.6	5460	4.9	50.1
**Lithuania**	154	1.2	85.82	280	2.2	84.9	411	1.5	81.1	6265	5.6	55.8
**Germany**	278	2.1	87.94	260	2.0	87.0	808	3.0	84.1	7213	6.5	62.3
**Netherlands**	20	0.2	88.09	18	0.1	87.1	72	0.3	84.3	671	0.6	62.9
**Denmark**	19	0.1	88.24	20	0.2	99.6	204	0.8	99.9	1037	0.9	98.6
**Czech republic**	NO	0.0	88.24	15	0.1	87.2	0	0.0	84.3	289	0.3	63.1
**Serbia**	-	0.0	88.24	6	0.0	87.3	-	0.0	84.3	96	0.1	63.2
**Slovenia**	NO	0.0	88.24	2	0.0	87.3	0	0.0	84.3	43	0.0	63.2
**Portugal**	NO	0.0	88.24	1	0.0	87.3	0	0.0	84.3	27	0.0	63.3
**Albania**	-	0.0	88.24	1	0.0	87.3	-	0.0	84.3	15	0.0	63.3
**Greece**	NO	0.0	88.24	1	0.0	87.3	0	0.0	84.3	27	0.0	63.3
**Spain**	NO	0.0	88.24	1	0.0	87.3	0	0.0	84.3	68	0.1	63.4
**Montenegro**	-	0.0	88.24	1	0.0	87.3	-	0.0	84.3	15	0.0	63.4
**Andorra**	-	0.0	88.24	0	0.0	87.3	-	0.0	84.3	1	0.0	63.4
**Luxembourg**	NO	0.0	88.24	0	0.0	87.3	0	0.0	84.3	8	0.0	63.4
**Slovakia**	NO	0.0	88.24	0	0.0	87.3	0	0.0	84.3	14	0.0	63.4
**North Macedonia**	-	0.0	88.24	0	0.0	87.3	-	0.0	84.3	1	0.0	63.4
**Liechtenstein**	NO	0.0	88.24	0	0.0	87.3	0	0.0	84.3	0	0.0	63.4
**Bosnia and Herzegovina**	-	0.0	88.24	0	0.0	87.3	-	0.0	84.3	10	0.0	63.4
**Italy**	NO	0.0	88.24	0	0.0	87.3	0	0.0	84.3	21	0.0	63.4
**Cyprus**	NO	0.0	88.24	0	0.0	87.3	0	0.0	84.3	0	0.0	63.4
**Iceland**	4	0.0	88.27	0	0.0	87.3	9	0.0	84.4	90	0.1	63.5
**Bulgaria**	NO	0	88.27	0	0.0	87.3	0	0.0	84.4	20	0.0	63.5
**Poland**	NA	0.0	88.27	864	6.7	94.0	849	3.1	87.5	18208	16.4	79.9
**United Kingdom**	451	3.4	91.71	305	2.4	96.4	461	1.7	89.2	3946	3.5	83.5
**Norway**	708	5.4	97.11	23	0.2	96.6	1483	5.5	94.6	3404	3.1	86.5
**Latvia**	379	2.9	100.00	263	2.0	98.6	1207	4.4	99.1	9130	8.2	94.7
**Hungary**	NO	0.0	100.00	40	0.3	98.9	62	0.2	99.3	1487	1.3	96.1
**Austria**	NO	0.0	100.00	40	0.3	99.2	0	0.0	99.3	867	0.8	96.8
**France**	NO	0.0	100.00	33	0.3	99.5	-42	-0.2	99.2	965	0.9	97.7
**Belgium**	NO	0.0	100.00	17	0.1	99.7	0	0.0	99.9	638	0.6	99.2
**Romania**	NO	0.0	100.00	11	0.1	99.8	24	0.1	100.0	424	0.4	99.6
**Switzerland**	0	0.0	100.00	11	0.1	99.9	1	0.0	100.0	265	0.2	99.8
**Croatia**	NO	0.0	100.00	10	0.1	100.0	0	0.0	100.0	171	0.2	100.0
**Albania**	-	0.0	100.00	1	0.0	100.0	-	0.0	100.0	15	0.0	100.0
**Total**	**13111**	**14.2**	**100.00**	**12918**	**15.1**	**100.00**	**27168**	**18.9**	**100.00**	**111209**	**100.0**	**100.00**

Table S.4 | Forest peatland area and emissions. *The absolute and cumulative fraction of total EU+ area and emissions of Forest Land on peat soils. The NIS area data from the countries above the green line roughly agree (±20%) with this research.*

	Forestland											
	NIS 2023			This Study			NIS 2023			This study		
Country	Area [kha]	%	%cum	Area [kha]	%	%cum	Emissions [kt]	%	%cum	Emissions [kt]	%	%cum
Finland	5963	45.5	45.48	5827	45.1	45.1	10653	39.2	39.2	18214	16.4	16.4
Sweden	4433	33.8	79.29	3897	30.2	75.3	6915	25.5	64.7	21997	19.8	36.2
Estonia	288	2.2	81.49	547	4.2	79.5	1052	3.9	68.5	10088	9.1	45.2
Ireland	414	3.2	84.65	422	3.3	82.8	2998	11.0	79.6	5460	4.9	50.1
Lithuania	154	1.2	85.82	280	2.2	84.9	411	1.5	81.1	6265	5.6	55.8
Germany	278	2.1	87.94	260	2.0	87.0	808	3.0	84.1	7213	6.5	62.3
Netherlands	20	0.2	88.09	18	0.1	87.1	72	0.3	84.3	671	0.6	62.9
Denmark	19	0.1	88.24	20	0.2	99.6	204	0.8	99.9	1037	0.9	98.6
Czech republic	NO	0.0	88.24	15	0.1	87.2	0	0.0	84.3	289	0.3	63.1
Serbia	-	0.0	88.24	6	0.0	87.3	-	0.0	84.3	96	0.1	63.2
Slovenia	NO	0.0	88.24	2	0.0	87.3	0	0.0	84.3	43	0.0	63.2
Portugal	NO	0.0	88.24	1	0.0	87.3	0	0.0	84.3	27	0.0	63.3
Albania	-	0.0	88.24	1	0.0	87.3	-	0.0	84.3	15	0.0	63.3
Greece	NO	0.0	88.24	1	0.0	87.3	0	0.0	84.3	27	0.0	63.3
Spain	NO	0.0	88.24	1	0.0	87.3	0	0.0	84.3	68	0.1	63.4
Montenegro	-	0.0	88.24	1	0.0	87.3	-	0.0	84.3	15	0.0	63.4
Andorra	-	0.0	88.24	0	0.0	87.3	-	0.0	84.3	1	0.0	63.4
Luxembourg	NO	0.0	88.24	0	0.0	87.3	0	0.0	84.3	8	0.0	63.4
Slovakia	NO	0.0	88.24	0	0.0	87.3	0	0.0	84.3	14	0.0	63.4
North Macedonia	-	0.0	88.24	0	0.0	87.3	-	0.0	84.3	1	0.0	63.4
Liechtenstein	NO	0.0	88.24	0	0.0	87.3	0	0.0	84.3	0	0.0	63.4
Bosnia and Herzegovina	-	0.0	88.24	0	0.0	87.3	-	0.0	84.3	10	0.0	63.4
Italy	NO	0.0	88.24	0	0.0	87.3	0	0.0	84.3	21	0.0	63.4
Cyprus	NO	0.0	88.24	0	0.0	87.3	0	0.0	84.3	0	0.0	63.4
Iceland	4	0.0	88.27	0	0.0	87.3	9	0.0	84.4	90	0.1	63.5
Bulgaria	NO	0	88.27	0	0.0	87.3	0	0.0	84.4	20	0.0	63.5
Poland	NA	0.0	88.27	864	6.7	94.0	849	3.1	87.5	18208	16.4	79.9
United Kingdom	451	3.4	91.71	305	2.4	96.4	461	1.7	89.2	3946	3.5	83.5
Norway	708	5.4	97.11	23	0.2	96.6	1483	5.5	94.6	3404	3.1	86.5
Latvia	379	2.9	100.00	263	2.0	98.6	1207	4.4	99.1	9130	8.2	94.7
Hungary	NO	0.0	100.00	40	0.3	98.9	62	0.2	99.3	1487	1.3	96.1
Austria	NO	0.0	100.00	40	0.3	99.2	0	0.0	99.3	867	0.8	96.8
France	NO	0.0	100.00	33	0.3	99.5	-42	-0.2	99.2	965	0.9	97.7
Belgium	NO	0.0	100.00	17	0.1	99.7	0	0.0	99.9	638	0.6	99.2
Romania	NO	0.0	100.00	11	0.1	99.8	24	0.1	100.0	424	0.4	99.6
Switzerland	0	0.0	100.00	11	0.1	99.9	1	0.0	100.0	265	0.2	99.8
Croatia	NO	0.0	100.00	10	0.1	100.0	0	0.0	100.0	171	0.2	100.0
Albania	-	0.0	100.00	1	0.0	100.0	-	0.0	100.0	15	0.0	100.0
**Total**	**13111**	**14.2**	**100.00**	**12918**	**15.1**	**100.00**	**27168**	**18.9**	**100.00**	**111209**	**100.0**	**100.00**

Reference for ‘own data’: These datasets have been developed over 10 years as part of the Global Peatland Database (GPD). We developed mapping approaches that link various science networks, methodologies and databases, including those of peatland/landscape ecology for understanding where and how peatlands may occur, those of remote sensing for identifying possible locations, and those of pedology (legacy soil maps) and (palaeo-)ecology for ground truthing. Such integration of old field data, specialized knowledge, and modern RS and GIS techniques enables acquiring comprehensive, detailed and relative reliable overview. The GPD is a living and rapidly growing collection of currently 50,000 publications (including books, congress proceedings, reprints, grey literature and maps) and countless digital files, legacy maps, and GIS datasets dealing directly and indirectly with peatland occurrence and condition worldwide and through the ages. A small part of the book collection is already accessible via the Peatland and Nature Conservation International Library (PeNCIL): https://greifswaldmoor.de/pencil-142.html

Major relevant publications supporting this work include Barthelmes et al. (2015), Joosten (2009), Joosten et al. (2017), and Tegetmeyer et al. (2024). The Global Peatland Map 2.0, incorporating the European Wetland Map, represents the most comprehensive and refined peatland dataset available, offering a new standard for understanding and managing peatlands worldwide.

Table S.5 | Sources of the geo-spatial data included in the Global Peatland Map 2.0 . *Datasets compiled and harmonized by the authors as part of the Global Peatland Database (GPD). The GPD is a living and expanding collection of >50,000 publications, legacy maps, and GIS datasets on peatland occurrence and condition worldwide. It integrates field data, specialized ecological knowledge, legacy soil maps, and modern RS/GIS techniques, providing the foundation for the Global Peatland Map 2.0.*

country	wetland category (peatland=p, wetland=w)	Reference
Andorra	p	Details in Tanneberger et al. 2017 (http://mires-and-peat.net/media/map19/map_19_22.pdf).
Andorra	w	Details in Tanneberger et al. 2017 (http://mires-and-peat.net/media/map19/map_19_22.pdf).
Albania	p	Details in Tanneberger et al. 2017 (http://mires-and-peat.net/media/map19/map_19_22.pdf).
Albania	p, w	OpenStreetMap. Natural: Wetland. https://wiki.openstreetmap.org/wiki/DE:Map_Features (accessed on 01 July 2024).
Albania	w	Wetlands of the Balkan Mediterranean territory (http://185.17.146.157/maps/67)
Austria	p	Details in Tanneberger et al. 2017 (http://mires-and-peat.net/media/map19/map_19_22.pdf)
Austria	w	Lazoski, W. Schwarz, U. (2011). Aueninventar Österreich: Bericht zur bundesweiten Übersicht der Auenobjekte, Wien.
Belgium	p, w	Decleer, K., Wouters J., Jacobs S., Staes J., Spanhove T., Meire P., van Diggelen, R. (2016). Mapping wetland loss and restoration potential in Flanders (Belgium): an ecosystem service perspective. Ecology and Society21(4), 46.
Belgium	p	Databank_Ondergrond_Vlaanderen_-_Vlaamse_overheid,_Departement_Omgeving,_VlaamsPlanbureau_voor_Omgeving_(VPO)_– (accessed_on_25.11.2022)_(https://www.dov.vlaanderen.be/)
Belgium	p	Carte des Principaux Types de Sols de Wallonie au 1/250000 constituée de polygones représentant les PTS. Géoportal de la Wallonie. https://geoportail.wallonie.be/home.html
Belgium	p, w	EIONET (2013) Member State reports on Art 17 reporting period 2007-2012. https://www.eionet.europa.eu/etcs/etc-be/activities/reporting/article-17/outcomes-2007-2012
Bosnia and Herzegovina	p	Details in Tanneberger et al. 2017 (http://mires-and-peat.net/media/map19/map_19_22.pdf) Details in Tanneberger et al. 2017 (Appendix 1; http://mires-and-peat.net/media/map19/map_19_22.pdf).
Bosnia and Herzegovina	w	EEA geospatial data catalogue (2012). Extended wetland ecosystem layer 2012 (raster100m) version 1, Nov. 2019.
Bulgaria	p	OpenStreetMap. Natural: Wetland. https://wiki.openstreetmap.org/wiki/DE:Map_Features (accessed on 24 June 2024).
Bulgaria	p, w	EEA geospatial data catalogue. Extended wetland ecosystem layer 2012 (raster 100m) version 1, Nov. 2019. https://sdi.eea.europa.eu/catalogue/idp/api/records/5fc1b45a-715a-466e-b576-1be0ced40e2a

country	wetland category (peatland=p, wetland=w)	Reference
Bulgaria	p	Koinov, V., Trashliev, H., Yolevski, M., Andonov, T., Ninov, N., Hadzhiyanakiev, A., Angelov, E., Boyadzhiev, T., Fotakieva, E., Krastanov, S., Staykov, Y. (1968). Soil map of Bulgaria at a scale of 1:400,000. GUGK, Sofia, Bulgaria.
Bulgaria	p	Details in Tanneberger et al. 2017 (http://mires-and-peat.net/media/map19/map_19_22.pdf)
Bulgaria	p	Michev, T. (ed.) (1995) National action plan for the conservation of the most importantwetlands in Bulgaria. Ministry of Environment, Sofia, Bulgaria, 55 p.
Croatia	p	Details in Tanneberger et al. 2017 (http://mires-and-peat.net/media/map19/map_19_22.pdf)
Croatia	p, w	OpenStreetMap. Natural: Wetland. https://wiki.openstreetmap.org/wiki/DE:Map_Features (accessed on 24 June 2024).
Croatia	w	EEA geospatial data catalogue. Extended wetland ecosystem layer 2012 (raster 100m) version 1, Nov. 2019. https://sdi.eea.europa.eu/catalogue/idp/api/records/5fc1b45a-715a-466e-b576-1be0ced40e2a
CzechRepublic	p	Details in Tanneberger et al. 2017 (http://mires-and-peat.net/media/map19/map_19_22.pdf) Details_in_Tanneberger_et_al._2017 (Appendix_1;_http://mires-and-peat.net/media/map19/map_19_22.pdf)
CzechRepublic	p	OpenStreetMap. Natural: Wetland. https://wiki.openstreetmap.org/wiki/DE:Map_Features (accessed on 24 June 2024).
CzechRepublic	w	EEA geospatial data catalogue. Extended wetland ecosystem layer 2012 (raster 100m) version 1, Nov. 2019. https://sdi.eea.europa.eu/catalogue/idp/api/records/5fc1b45a-715a-466e-b576-1be0ced40e2a
CzechRepublic	w	OpenStreetMap. Natural: Wetland. https://wiki.openstreetmap.org/wiki/DE:Map_Features (accessed on 24 June 2024).
Denmark	p	Skov- og Naturstyrelsen (2009) *Naturbeskyttelsesloven, Lovbekendtgørelse nr. 933 af24.09.2009 om naturbeskyttelse.* [Nature Protection Act, Act no. 933 of 24.09.2009 on nature protection] (in Danish)
Denmark	p	K Adhikari, RB Kheir, MB Greve, PK Bøcher, BP Malone, B Minasny, AB McBratney, M.H. Greve (2013) High-resolution 3-D mapping of soil texture in Denmark. Soil Science Society of America Journal, Wiley Online Library
Denmark	w	EEA geospatial data catalogue. Extended wetland ecosystem layer 2012 (raster 100m) version 1, Nov. 2019. https://sdi.eea.europa.eu/catalogue/idp/api/records/5fc1b45a-715a-466e-b576-1be0ced40e2a
Estonia	p	Kmoch, A. 2017. Soil map of Estonia – Mullastikukaart. National Soilmap of Estonia,1:10,000, dataset deposit (https://datadoi.ee/handle/33/103?show=full)
Estonia	p	Estonian Topographic Database, Map data: Estonian Land Board 06.03.2023. https://geoportaal.maaamet.ee/eng/spatial-data/estonian-topographic-database-p305.html

country	wetland category (peatland=p, wetland=w)	Reference
Finland	p	Middleton, M., Laatikainen, M., Kivilompolo, J., Harju, A., Lerssi, J., Valkama, M., Pitkänen, T., Pohjankukka, J., Balazs, A., Tuominen, S., Zelioli, L., Farahnakian, F., Nevalainen, P., Heikkonen. J., (2023). GeologicalSurvey_of_Finland_Information Solutions/Environmental_Solutions_(2023)._Technical_descriptionfor_the_peatland_site_type_data_of_Finland._Report_73.
France	p	Peatland GIS data developed under ALFAwetlands project for the Spain catchment. Contact: Adrià Descals (adriadescals@gmail.com)
France	p	Gilbert, D., Muller, F., Bernard, G., Pilloix, M. (2021). Digitized inventory of the Frenchpeatlands of 1949. Laboratoire Chrono-environnement (UMR 6249), doi:10.25666/DATAOSU-2021-03-01
France	p	Details in Tanneberger et al. 2017 (http://mires-and-peat.net/media/map19/map_19_22.pdf) Details in Tanneberger et al. 2017 (Appendix 1; http://mires-and-peat.net/media/map19/map_19_22.pdf)
France	p	OpenStreetMap. Natural: Wetland. https://wiki.openstreetmap.org/wiki/DE:Map_Features (accessed on 24 June 2024).
France	w	OpenStreetMap. Natural: Wetland. https://wiki.openstreetmap.org/wiki/DE:Map_Features (accessed on 24 June 2024).
France	w	EEA geospatial data catalogue. Extended wetland ecosystem layer 2012 (raster 100m) version 1, Nov. 2019. https://sdi.eea.europa.eu/catalogue/idp/api/records/5fc1b45a-715a-466e-b576-1be0ced40e2a
Germany	p	Tegetmeyer, C., Barthelmes, K.-D., Busse, S. & Barthelmes, A. (2020). Aggregierte Karte derorganischen Böden Deutschlands (pdf). Greifswald Moor Centrum-Schriftenreihe 01/2020 (Selbstverlag, ISSN 2627-910X),10 S.
Germany	p	Wittnebel, M., Frank, S., Tiemeyer, B. (2023). Aktualisierte Kulisse organischer Böden in Deutschland. [Datensatz], OpenAgrar-Repository, Göttingen, Germany. (https://atlas.thuenen.de/layers/geonode_data:geonode:ti_kulisse_kat_final_v10)
Germany	w	OpenStreetMap. Natural: Wetland. https://wiki.openstreetmap.org/wiki/DE:Map_Features(accessed on 24 June 2024).
Greece	p	Details in Tanneberger et al. 2017 (http://mires-and-peat.net/media/map19/map_19_22.pdf)
Greece	p, w	Wetlands of the Balkan Mediterranean territory. Fitoka Eleni, Petrov P, Ivanova S., Hatziirdonou L, Terziyska I. (editors). 2020. The Regional Balkan - Mediterranean Wetland Mapping & Connectivity Assessment: methodological approach, findings, future considerations. WetMainAreas INTERREG Balkan - Mediterranean project TechnicalPublication. 53 pages.
Hungary	p	Pásztor, L., Laborczia, A., Bakacsi, Z., Szabó, J., Illés, G. (2018). Compilation of a nationalsoil-type map for Hungary by sequential classification methods, *Geoderma* 311, 93-108
Hungary	p	Várallyay, Y, Szucs, L., Rajkai, K., Zilahy, P., Murányi, A. (1980). Soil_water_managementcategories_of_Hungarian_soils_and_the_map of_soil_water properties (of 1:100,000). Agrokémia és Talajtan 29: 77-112. (*in Hungarian*).

country	wetland category (peatland=p, wetland=w)	Reference
Hungary	p, w	Ministry of Agriculture (2019). Development of an ecosystem basemap and data model.https://www.interregeurope.eu/good-practices/national-ecosystem-map-of-hungary
Iceland	p	Guðmundsson, J., Brink, S.H. (2014). IGLUD - Icelandic Geographical Land Use Database, Agricultural University of Iceland, Reykjavík, Iceland.
Iceland	w	EEA geospatial data catalogue. Extended wetland ecosystem layer 2012 (raster 100m) version 1, Nov. 2019. https://sdi.eea.europa.eu/catalogue/idp/api/records/5fc1b45a-715a-466e-b576-1be0ced40e2a
Ireland	w	EEA geospatial data catalogue. Extended wetland ecosystem layer 2012 (raster 100m) version 1, Nov. 2019. https://sdi.eea.europa.eu/catalogue/idp/api/records/5fc1b45a-715a-466e-b576-1be0ced40e2a
Ireland	p	Gilet, L., Morley, T. R., Flynn, R., Connolly, J. (2024). An adaptive mapping framework forthe management of peat soils: A New Irish peat soils map. Geoderma 447, 116933.
Italy	p	Details in Tanneberger et al. 2017 (http://mires-and-peat.net/media/map19/map_19_22.pdf)
Italy	p	Gardin L., Chiesi M., Fibbi L., Maselli F. (2021) Mapping soil organic carbon in Tuscanythrough the statistical combination of ground observations with ancillary and remote sensing data, *Geoderma* 404, 115386.
Italy	p	OpenStreetMap. Natural: Wetland. https://wiki.openstreetmap.org/wiki/DE:Map_Features (accessed on 13 June 2024).
Italy	w	OpenStreetMap. Natural: Wetland. https://wiki.openstreetmap.org/wiki/DE:Map_Features (accessed on 13 June 2024).
Latvia	p	Ivanovs, J., Haberl, A., Melniks, R. (2024). Modeling Geospatial Distribution of Peat Layer Thickness Using Machine Learning and Aerial Laser Scanning Data. Land 13, 466. https://doi.org/10.3390/land13040466
Latvia	p	Šnore, A. (2013). Kūdras ieguve. [Extraction of peat]. NORDIK, Riga, Latvia, 432 p. (inLatvian).
Latvia	w	EEA geospatial data catalogue. Extended wetland ecosystem layer 2012 (raster 100m) version 1, Nov. 2019. https://sdi.eea.europa.eu/catalogue/idp/api/records/5fc1b45a-715a-466e-b576-1be0ced40e2a
Liechtenstein	p	Global Peatland Database 2024 (own developed data).
Liechtenstein	w	EEA geospatial data catalogue. Extended wetland ecosystem layer 2012 (raster 100m) version 1, Nov. 2019. https://sdi.eea.europa.eu/catalogue/idp/api/records/5fc1b45a-715a-466e-b576-1be0ced40e2a

country	wetland category (peatland=p, wetland=w)	Reference
Lithuania	p	Lithuania fund for Nature and Lithuanian Geological Survey (2005). Lietuvos durpynų irpelkių žemėlapis. [Map of peatlands and mires in Lithuania]. scale 1:200,000, GIS dataset.(https://www.geoportal.lt/geoportal/subscribe/-/asset_publisher/I0YH9ZsWns4x/content/nauji-duomenys-geoportal-lt-lietuvos-pelkiu-ir-durpynu-duomenu-perziuros-paslauga)
Lithuania	p	Lietuvos pelkių ir durpynų GIS duomenų bazės atnaujinimas. VšĮ Gamtos paveldo fondas,2018
Luxembourg	p	Details in Tanneberger et al. 2017 (http://mires-and-peat.net/media/map19/map_19_22.pdf) Details_in_Tanneberger_et_al._2017 (Appendix_1;_http://mires-and-peat.net/media/map19/map_19_22.pdf)
Luxembourg	w	EEA geospatial data catalogue. Extended wetland ecosystem layer 2012 (raster 100m) version 1, Nov. 2019. https://sdi.eea.europa.eu/catalogue/idp/api/records/5fc1b45a-715a-466e-b576-1be0ced40e2a
Montenegro	p	Details in Tanneberger et al. 2017 (Appendix 1; http://mires-and-peat.net/media/map19/map_19_22.pdf)
Montenegro	w	EEA geospatial data catalogue. Extended wetland ecosystem layer 2012 (raster 100m) version 1, Nov. 2019. https://sdi.eea.europa.eu/catalogue/idp/api/records/5fc1b45a-715a-466e-b576-1be0ced40e2a
Netherlands	p	Wosten, H, F., de Vries, T., Hoogland, H., Massop, A., Veldhuizen, H., Vroon, J.,Wesseling, J. Heijkers, Bolman, A. (2012). Bofek2012 - de nieuwe bodemfysischeschematisatie van Nederland. Alterra, Wageningen, Rapport 2387. (http://edepot.wur.nl/247678)
North Macedonia	p, w	Marija Chobanova unpublished
North Macedonia	w	Wetlands of the Balkan Mediterranean territory, Fitoka Eleni, Petrov P, Ivanova S., Hatziirdonou L, Terziyska I. (editors). 2020. TheRegional Balkan - Mediterranean Wetland Mapping & Connectivity Assessment: methodological approach, findings, future considerations. WetMainAreas INTERREG Balkan - Mediterranean project TechnicalPublication. 53 pages.
Norway	p	Details in Tanneberger et al. 2017 (http://mires-and-peat.net/media/map19/map_19_22.pdf) Tanneberger et al 2017. The peatland Map of Europe. Mires and Peat 19 Artikle 22 1-8
Norway	p	NIBIO (2024). Arealressurskart – AR50, type 60 (organic soils), national coverage. Norwegian Institute of Bioeconomy Research (NIBIO). Available at: https://kart8.nibio.no/nedlasting/dashboard
Poland	p, w	Ministerstwo Środowiska (2006). System Informacji Przestrzennej o Mokradłach Polski.Instytut Melioracji i Użytków Zielonych w Falentach, Zakład Ochrony Przyrody Obszarów Wiejskich.

country	wetland category (peatland=p, wetland=w)	Reference
Poland	p, w	Lasy Państwowe (2020). Bank Danych o Lasach, https://www.bdl.lasy.gov.pl (accessed inJuly 2021).<br>KZGW (2010). Mapa Podziału Hydrograficznego Polski. 1:10,000, (jeziora, rzeki_s), KrajowyZarząd Gospodarki Wodnej, Warszawa https://dane.gov.pl/pl/dataset/2167,mapa-podzialu-hydrograficznego-polski-w-skali-110(accessed in October 2021).
Portugal	p	van Giersbergen, Q. (2022). Mapping European Greenhouse gas emissions of drainedpeatlands. Master Thesis. Wageningen University, the Netherlands.
Portugal	p, w	OpenStreetMap. Natural: Wetland. https://wiki.openstreetmap.org/wiki/DE:Map_Features OpenStreetMap. Natural: Wetland. https://wiki.openstreetmap.org/wiki/DE:Map_Features (accessed on 17 June 2024).
Portugal	w	EEA geospatial data catalogue. Extended wetland ecosystem layer 2012 (raster 100m) version 1, Nov. 2019. https://sdi.eea.europa.eu/catalogue/idp/api/records/5fc1b45a-715a-466e-b576-1be0ced40e2a
Romania	w	EEA geospatial data catalogue. Extended wetland ecosystem layer 2012 (raster 100m) version 1, Nov. 2019. https://sdi.eea.europa.eu/catalogue/idp/api/records/5fc1b45a-715a-466e-b576-1be0ced40e2a
Romania	p, w	OpenStreetMap. Natural: Wetland. https://wiki.openstreetmap.org/wiki/DE:Map_Features OpenStreetMap. Natural: Wetland. https://wiki.openstreetmap.org/wiki/DE:Map_Features (accessed on 27 May 2024).
Serbia	p	Details in Tanneberger et al. 2017 (http://mires-and-peat.net/media/map19/map_19_22.pdf) Details in Tanneberger et al. 2017 (Appendix 1; http://mires-and-peat.net/media/map19/map_19_22.pdf)
Serbia	w	EEA geospatial data catalogue. Extended wetland ecosystem layer 2012 (raster 100m) version 1, Nov. 2019. https://sdi.eea.europa.eu/catalogue/idp/api/records/5fc1b45a-715a-466e-b576-1be0ced40e2a
Slovakia	p	Details in Tanneberger et al. 2017 (http://mires-and-peat.net/media/map19/map_19_22.pdf) Details_in_Tanneberger_et_al._2017 (Appendix_1;_http://mires-and-peat.net/media/map19/map_19_22.pdf)
Slovakia	w	Miklos, L. (2002). Landscape Atlas of the Slovak Republic. Slovakia: Ministry of Environment of the Slovak Republic, Slovak Environmental Agency Banska Bystrica. https://inis.iaea.org/search/search.aspx?orig_q=RN:34041334
Slovakia	p, w	OpenStreetMap. Natural: Wetland. https://wiki.openstreetmap.org/wiki/DE:Map_Features (accessed on 27 May 2024).
Slovakia	w	EEA geospatial data catalogue. Extended wetland ecosystem layer 2012 (raster 100m) version 1, Nov. 2019. https://sdi.eea.europa.eu/catalogue/idp/api/records/5fc1b45a-715a-466e-b576-1be0ced40e2a
Slovenia	p	Details in Tanneberger et al. 2017 (http://mires-and-peat.net/media/map19/map_19_22.pdf) Details in Tanneberger et al. 2017 (Appendix_1;_http://mires-and-peat.net/media/map19/map_19_22.pdf)

country	wetland category (peatland=p, wetland=w)	Reference
Slovenia	p	Republika Slovenia, Ministrstvo za kmetijstvo, gozdarstvo in prehrano (2019). Update ofthe pedological map (pedological profiles) Coordinate System: D96/TM https://www.gov.si/
Slovenia	w	EEA geospatial data catalogue. Extended wetland ecosystem layer 2012 (raster 100m) version 1, Nov. 2019. https://sdi.eea.europa.eu/catalogue/idp/api/records/5fc1b45a-715a-466e-b576-1be0ced40e2a
Spain	p	Adrià Descals Ferrando (2024), CREAF, https://www.creaf.cat/en, unpublished product ofthe AlfaWetlands project
Spain	p, w	OpenStreetMap. Natural: Wetland. https://wiki.openstreetmap.org/wiki/DE:Map_Features (accessed on 14 June 2024).
Spain	w	EEA geospatial data catalogue. Extended wetland ecosystem layer 2012 (raster 100m) version 1, Nov. 2019. https://sdi.eea.europa.eu/catalogue/idp/api/records/5fc1b45a-715a-466e-b576-1be0ced40e2a
Sweden	p	Rimondini, I., Hugelius, G., Gumbricht, T., Ahlström, A. (2023). Maps of peatlands in the forested landscape of Sweden. Dataset version 1. Bolin Centre Database. https://doi.org/10.17043/rimondini-2023-peatlands-2
Switzerland	p	Details in Tanneberger et al. 2017 (http://mires-and-peat.net/media/map19/map_19_22.pdf) Details in Tanneberger et al. 2017 (Appendix 1; http://mires-and-peat.net/media/map19/map_19_22.pdf)
Switzerland	p	Details in Tanneberger et al. 2017 (http://mires-and-peat.net/media/map19/map_19_22.pdf) Details in Tanneberger et al. 2017 (Appendix 1; http://mires-and-peat.net/media/map19/map_19_22.pdf)
Switzerland	p	Wüst-Galley, C., Grünig, A., Leifeld, J. (2015). Locating Organic Soils for the Swiss Greenhouse Gas Inventory. Agroscope Science, No. 26, Agroscope, Zürich.
Switzerland	w	EEA geospatial data catalogue. Extended wetland ecosystem layer 2012 (raster 100m) version 1, Nov. 2019. https://sdi.eea.europa.eu/catalogue/idp/api/records/5fc1b45a-715a-466e-b576-1be0ced40e2a
UK_England	p	Natural England (ARM team) (2008). National Soils map. Actualized 2022. BGS, CranfieldUniversity (NSRI) and OS. https://naturalengland-defra.opendata.arcgis.com/search?categories=%252Fcategories%252Fsoil
UK_North Ireland	p	Evans, C., Artz, R., Moxley, J., Smyth, M-A., Taylor, E., Archer, N., Burden, A., Williamson,J. , Donnelly, D., Thomson, A., Buys, G., Malcolm, H., Wilson, D., Renou-Wilson, F., PottsJ. (2017). Implementation of an emission inventory for UK peatlands. Report to the Department for Business, Energy and Industrial Strategy, Centre for Ecology and Hydrology, Bangor, 88pp.

country	wetland category (peatland=p, wetland=w)	Reference
UK_Scotland	p	The Macaulay Land Use Reasearch Institute (1993). The Land Cover of Scotland 1988.The Macaulay Land Use Reasearch Institute, Craigiebuckler Aberdeen AB92QJ. Copyright Scottish Government, contains Ordnance Survey data © Crown copyright and database right (1988). https://www.hutton.ac.uk/sites/default/files/files/soils/lcs88_executive_summary.pdf
UK_Wales	p	Evans, C.D., Scholefeld, P., Rawlins, B., Grebby, S., Jones, P., Williamson, J.M. (2020). Unified peat map for Wales. NERC Environmental Information Data Centre, dataset. https://doi.org/10.5285/58139ce6-63f9-4444-9f77-fc7b5dcc00d8
UK	w	EEA geospatial data catalogue. Extended wetland ecosystem layer 2012 (raster 100m)v ersion 1, Nov. 2019. https://sdi.eea.europa.eu/catalogue/idp/api/records/5fc1b45a-715a-466e-b576-1be0ced40e2a

Overlaying the land use map for peatlands with this productivity map resulted in the mean and standard deviation productivity index per country as shown in Table S1. Here only for the countries where information in the NIR reporting was available about the deviation of shallow and deep where evaluated. The mean productivity index (PI) for grassland is 6.321 and for forestland 6.079. It can be observed that the PI for countries in colder climates (towards boreal) have a lower PI than the countries towards more temperate climatic conditions.

When applying the mean grassland PI value to distinguish if a peat grassland is shallow drained (below 6.321 PI) or deep drained (above 6.321 PI), 8 out of 13 countries listed above in Table 3 where estimated good (within a ±5% range) to the NIR reporting deviation of deep/shallow drained grassland (Martin & Couwenberg, 2021). When applying -1 standard deviation of the mean (4.915 PI) as the threshold for grassland only Finland and Ireland were correctly estimated according to the NIR reporting (Martin & Couwenberg, 2021). Therefore, in this research the mean PI value of the productivity map developed by JRC in 2013 is assumed to be a quite good estimator of when a grassland is deep or shallow drained. Because there is no EU wide information available about the deviation of nutrient rich and nutrient poor forestland and wetlands this research used the same method (mean of the 13 listed countries above) as the threshold value. Forestland and wetlands this value was set to 6.079 PI

Table S.6 | Productivity index per country. *Mean productivity index (PI) and standard deviation for grassland and forest land on peat soils, derived from overlaying the JRC productivity map (Tóth et al., 2013) with the peatland land-use map. These values were used to distinguish shallow versus deep drained conditions.*

	Grassland		*Forestland*	
Countries	*Mean (PI)*	*Std.dev (PI)*	*Mean (PI)*	*Std.dev (PI)*
Sweden	*4.13*	*2.25*	*5.93*	*0.81*
Lithuania	*5.01*	*1.07*	*4.86*	*0.74*
Hungary	*5.48*	*0.86*	*5.43*	*0.75*
Finland	*5.56*	*1.12*	*5.00*	*1.84*
France	*5.91*	*1.78*	*6.26*	*1.56*
Slovakia	*5.96*	*0.69*	*5.78*	*0.97*
Austria	*6.01*	*1.55*	*6.10*	*1.40*
Ireland	*6.52*	*2.25*	*6.49*	*2.04*
Czech	*6.73*	*0.51*	*6.69*	*0.24*
Denmark	*6.98*	*1.14*	*6.75*	*1.11*
Germany	*7.20*	*1.24*	*6.86*	*1.02*
Netherlands	*8.01*	*1.65*	*7.16*	*1.54*
Latvia	*8.67*	*2.08*	*5.74*	*1.06*
Average	*6.32*	*1.41*	*6.08*	*1.16*

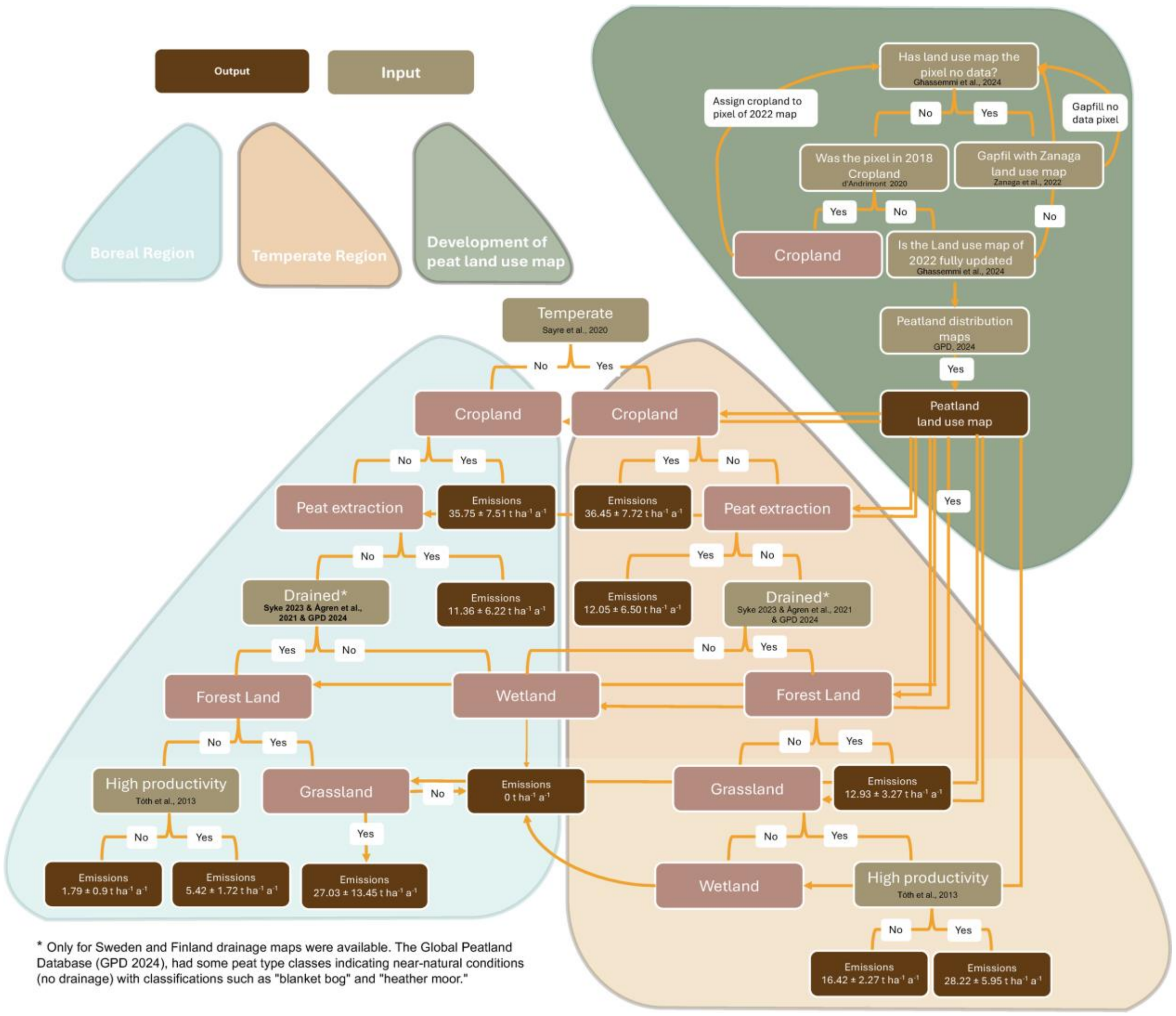

Figure S.1 | Workflow for peatland land-use and emission mapping. *Schematic overview of the method for developing the peatland land use map and the peatland emission map. Showing which input data has been used and which emission factor has been applied and in which order. First the peatland land use has been developed that serves as input for the emissions map.*

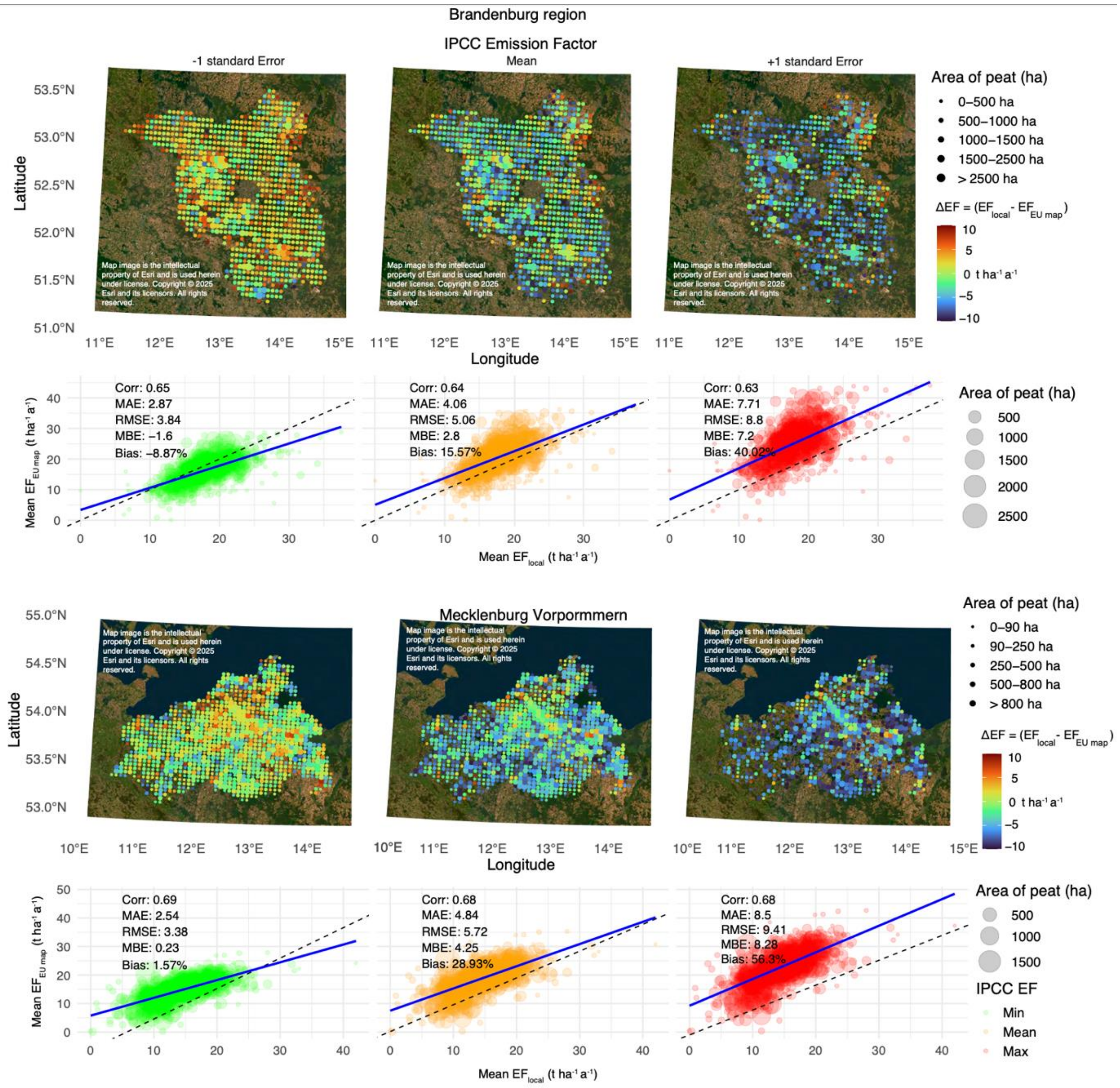

***Figure S.2* | Comparison with regional peatland emission maps.** The difference (per 5x5 km^2) between the EU emission map developed in this study and the local spatial peatland emission map for the Brandenburg (N=1750) and Mecklenburg Vorpommern (N=1930) regions in Germany. Red colour indicates lower mean emission factor (EF) for that area in the EU map and blue indicates higher EF estimates in the EU map. The point cloud shows the 1 to 1 relation (dashed line) and the trend (blue line) and the EF per point whereas the size indicates the cumulative peatland area. Sources: (Reichelt, F. 2021; Reichelt, F. 2024). Additionally, the impact of the low IPCC EF estimate (-1 standard deviation; left column) vs. the high estimate (+1 standard deviation; right column) is shown spatially and 1 to 1 relation. For correlation we assumed linearity and used a Pearson corelation

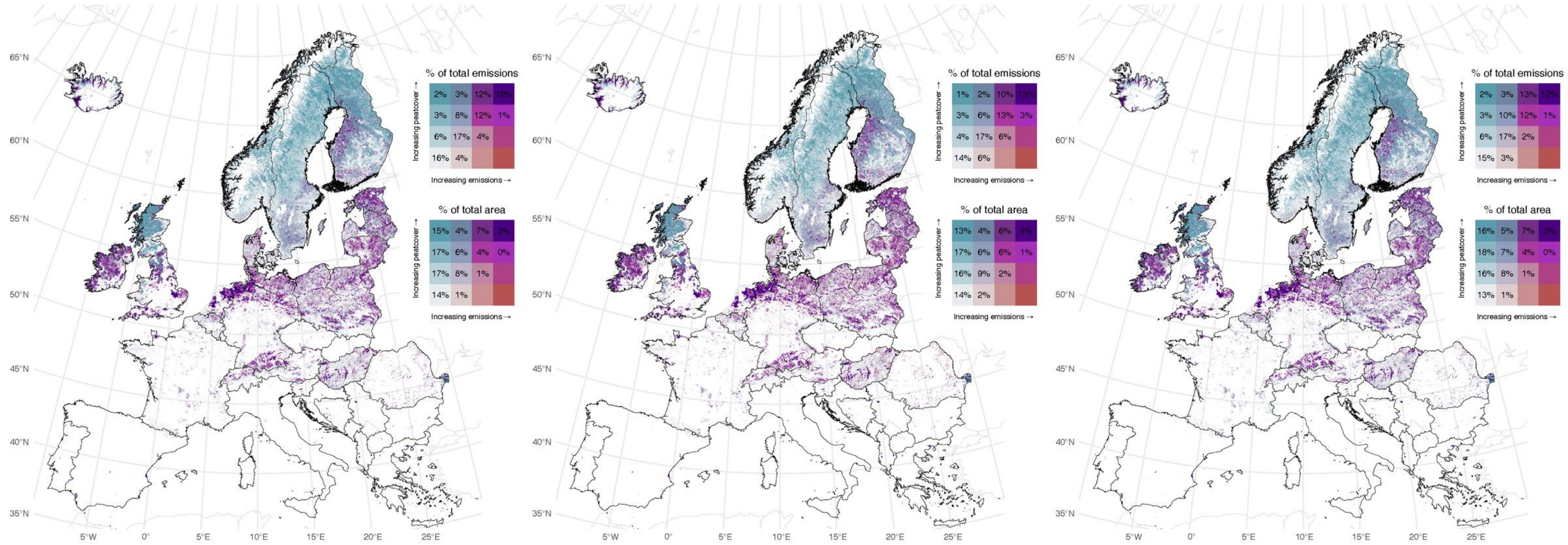

Figure S.3 | Uncertainty in peatland emission hotspot estimates. From left to right -1 standard error of the IPCC emission factor, mean IPCC emission factor, and +1 standard error of the IPCC emission factor applied per pixel (100 m^2) and downscaled to 1 km^2 for better visualisation. This shows that the major hotspots do not significantly change even though the standard error between EF differs. Only the contributions of certain colours to the total emissions and area changes
