# Supplementary material for: Identifying hotspots of greenhouse gas emissions from drained peatlands in the European Union
Source: Nat Commun. 2025 Dec 2;16:10825. doi: 10.1038/s41467-025-65841-6 (PMC12673095; doi:10.1038/s41467-025-65841-6)
Supplement: Supplementary file 2 — Reporting Summary [file 41467_2025_65841_MOESM2_ESM.pdf]

nature portfolio

Corresponding author(s): Quint van Giersbergen

Last updated by author(s): Sep 4, 2025

Reporting Summary

Nature Portfolio wishes to improve the reproducibility of the work that we publish. This form provides structure for consistency and transparency in reporting. For further information on Nature Portfolio policies, see our Editorial Policies and the Editorial Policy Checklist.

Statistics

For all statistical analyses, confirm that the following items are present in the figure legend, table legend, main text, or Methods section.

n/a	Confirmed	
[x]	[ ]	The exact sample size (*n*) for each experimental group/condition, given as a discrete number and unit of measurement
[x]	[ ]	A statement on whether measurements were taken from distinct samples or whether the same sample was measured repeatedly
[x]	[ ]	The statistical test(s) used AND whether they are one- or two-sided<br>*Only common tests should be described solely by name; describe more complex techniques in the Methods section.*
[x]	[ ]	A description of all covariates tested
[ ]	[x]	A description of any assumptions or corrections, such as tests of normality and adjustment for multiple comparisons
[ ]	[x]	A full description of the statistical parameters including central tendency (e.g. means) or other basic estimates (e.g. regression coefficient) AND variation (e.g. standard deviation) or associated estimates of uncertainty (e.g. confidence intervals)
[x]	[ ]	For null hypothesis testing, the test statistic (e.g. *F*, *t*, *r*) with confidence intervals, effect sizes, degrees of freedom and *P* value noted<br>*Give P values as exact values whenever suitable.*
[x]	[ ]	For Bayesian analysis, information on the choice of priors and Markov chain Monte Carlo settings
[x]	[ ]	For hierarchical and complex designs, identification of the appropriate level for tests and full reporting of outcomes
[ ]	[x]	Estimates of effect sizes (e.g. Cohen's *d*, Pearson's *r*), indicating how they were calculated

Our web collection on statistics for biologists contains articles on many of the points above.

Software and code

Policy information about availability of computer code

Data collection	The research relied on compiling and harmonising existing datasets that had already been collected by others. Google (Scholar and search) was used.
Data analysis	We analysed the data using R version 4.4.1 (R Core Team, 2024). Analyses relied on open-source R packages, including biscale (v1.0.0) for hotspot mapping and standard spatial/statistical packages for data handling. No commercial or custom software beyond the R scripts provided on Zenodo (https://zenodo.org/records/14974022) was used.

For manuscripts utilizing custom algorithms or software that are central to the research but not yet described in published literature, software must be made available to editors and reviewers. We strongly encourage code deposition in a community repository (e.g. GitHub). See the Nature Portfolio guidelines for submitting code & software for further information.

Data

Policy information about availability of data

All manuscripts must include a data availability statement. This statement should provide the following information, where applicable:

- Accession codes, unique identifiers, or web links for publicly available datasets
- A description of any restrictions on data availability
- For clinical datasets or third party data, please ensure that the statement adheres to our policy

All datasets used in this study are publicly available. The European Wetland Map (Tegetmeyer et al., 2025) is accessible via Zenodo: https://doi.org/10.5281/zenodo.14883520. EU crop maps are available from d'Andrimont et al., 2021: https://doi.org/10.1016/j.rse.2021.112708 and Ghassemi et al., 2024: https://

doi.org/10.1038/s41597-024-03884-y. The land use map of Witjes et al. (2022) can be accessed through Zenodo: https://doi.org/10.5281/zenodo.4725429. The biomass productivity map (Tóth et al., 2013) is available at Ecological Processes: https://doi.org/10.1186/2192-1709-2-32. The Köppen-Geiger climate classification (Beck et al., 2023) is accessible at Scientific Data: https://doi.org/10.1038/s41597-023-02549-6. Processed data supporting the findings of this study are available from the corresponding author upon reasonable request. The scripts used to analyse the data and generate the results are available via Zenodo: https://zenodo.org/records/14974022

Research involving human participants, their data, or biological material

Policy information about studies with human participants or human data. See also policy information about sex, gender (identity/presentation), and sexual orientation and race, ethnicity and racism.

Reporting on sex and gender	N/A
Reporting on race, ethnicity, or other socially relevant groupings	N/A
Population characteristics	N/A
Recruitment	N/A
Ethics oversight	N/A

Note that full information on the approval of the study protocol must also be provided in the manuscript.

Field-specific reporting

Please select the one below that is the best fit for your research. If you are not sure, read the appropriate sections before making your selection.

☐ Life sciences ☐ Behavioural & social sciences ☒ Ecological, evolutionary & environmental sciences

For a reference copy of the document with all sections, see nature.com/documents/nr-reporting-summary-flat.pdf

Ecological, evolutionary & environmental sciences study design

All studies must disclose on these points even when the disclosure is negative.

Study description	This study mapped greenhouse gas (GHG) emissions from drained peatlands across the European Union (EU+). Using high-resolution peatland and land-use maps (10–30 m), IPCC (2014) emission factors stratified by climate zone, land use, nutrient and drainage status, and biomass productivity indices, emissions were estimated at ~10 m resolution and aggregated to 1 km^2. Comparisons were made with National Inventory Submissions (NIS) and regional maps (Germany) for validation. Results showed total EU peatland emissions of 232 ± 56 Mt CO_2e yr^{-1}, nearly double reported NIS values, with four major hotspot regions (North Sea area, eastern Germany, Baltics/eastern Poland, and central Ireland). The study highlights systematic under-reporting in national inventories and provides the first EU-wide spatial hotspot map to guide peatland rewetting and climate policy.
Research sample	This study used existing spatial datasets to represent peatlands across the European Union and associated countries (EU+). The research sample consisted of geospatial layers on peat and peaty soils from the European Wetland Map (Tegetmeyer et al., 2025), stratified by land use using EU crop maps (d'Andrimont et al., 2021; Ghassemi et al., 2024) and complemented by additional land cover data (Witjes et al., 2022). Emission factors were applied following IPCC (2014) guidelines, with nutrient and drainage status inferred from a European biomass productivity map (Tóth et al., 2013). This sample choice allowed for harmonized, large-scale coverage of peatland extent and condition across Europe, representing the entire EU+ peatland population.
Sampling strategy	We only want to include the most recent data and of highest spatial resolution as possible. Therefore older peatland maps and or landuse maps where not used.
Data collection	Using connections with other researchers and using google (scholar and search)
Timing and spatial scale	Data collection was performed between 2021 and 2025, where the spatial data originated from different periods, peatland map 2025, land use images from 2018 and 2022 and the biomass map from 2012.
Data exclusions	Only old and low resolution maps where excluded if a higher resolution and or newer map was available
Reproducibility	All analyses in this study were conducted using openly available datasets, harmonized processing steps, and standardized emission factors (IPCC, 2014) to ensure reproducibility. The full scripts have been made available via Zenodo (https://zenodo.org/records/14974022), allowing independent replication of the spatial analyses and hotspot identification. Validation was carried out by comparing results with National Inventory Submissions (UNFCCC, 2023) and two independent regional peatland emission maps (Brandenburg and Mecklenburg-Vorpommern), both of which showed good agreement in mean emissions and spatial patterns. No failed replication attempts were encountered;
Randomization	N/A only 1 sample size
Blinding	N/A

Did the study involve field work? ☐ Yes ☒ No

Reporting for specific materials, systems and methods

We require information from authors about some types of materials, experimental systems and methods used in many studies. Here, indicate whether each material, system or method listed is relevant to your study. If you are not sure if a list item applies to your research, read the appropriate section before selecting a response.

Materials & experimental systems

n/a	Involved in the study
☒	☐ Antibodies
☒	☐ Eukaryotic cell lines
☒	☐ Palaeontology and archaeology
☒	☐ Animals and other organisms
☒	☐ Clinical data
☒	☐ Dual use research of concern
☒	☐ Plants

Methods

n/a	Involved in the study
☒	☐ ChIP-seq
☒	☐ Flow cytometry
☒	☐ MRI-based neuroimaging

Plants

Seed stocks	NA
Novel plant genotypes	NA
Authentication	NA
